# Risks of autoimmune and inflammatory post-acute COVID-19 conditions: a network cohort study in six European countries, the USA and Korea

**DOI:** 10.1136/bmjph-2024-001686

**Published:** 2026-07-24

**Authors:** Theresa Ms Burkard, Kim López-Güell, Martí Català, Edward Burn, Antonella Delmestri, Sara Khalid, Annika M Jödicke, Daniel Dedman, Jessie Oyinlola, Alicia Abellan, Laura Pérez-Crespo, Núria Mercadé-Besora, Talita Duarte-Salles, Daniel Prieto-Alhambra, Johnmary Arinze, Mees Mosseveld, Raivo Kolde, Jaime Meléndez, Raúl López-Blasco, Álvaro Martínez, Bernardo Valdivieso, Dominique Delseny, Gregoire Mercier, Chungsoo Kim, Ji-Woo Kim, Kristin Kostka, Juan Manuel Ramírez-Anguita, Miguel Angel Mayer, Nhung T H Trinh, Hedvig Nordeng, Rogersyp Paredes, Anneli Uusküla, Akihiko Nishimura, Cora Loste, Lourdes Mateu, Junqing Xie

**Affiliations:** 1 Nuffield Department of Orthopaedics, Rheumatology and Musculoskeletal Sciences, University of Oxford, Oxford, UK; 2 CPRD, Medicines and Healthcare Products Regulatory Agency, London, UK; 3 IDIAP Jordi Gol, Barcelona, Spain; 4 Department of Medical Informatics, Erasmus Medical Center, Rotterdam, The Netherlands; 5 Institute of Computer Science, University of Tartu, Tartu, Estonia; 6 Aragon Health Sciences Institute, Zaragoza, Spain; 7 Instituto de Investigación Sanitaria La Fe, Valencia, Spain; 8 Hospital Universitari i Politecnic La Fe, Valencia, Spain; 9 Public Health Department, Centre Hospitalier Universitaire de Montpellier, Montpellier, France; 10 Universite de Montpellier, Montpellier, France; 11 Department of Biomedical Sciences, Ajou University, Suwon, Korea (the Republic of); 12 Big Data Department, Health Insurance Review and Assessment Service, Wonju, Korea (the Republic of); 13 Northeastern University - The Roux Institute, Portland, Maine, USA; 14 Hospital del Mar Medical Research Institute, Barcelona, Spain; 15 Department of Pharmacy, University of Oslo, Oslo, Norway; 16 Department of Child Health and Development, Norwegian Institute of Public Health, Oslo, Norway; 17 Infectious Diseases Department, Hospital Universitari Germans Trias i Pujol, Badalona, Spain; 18 Department of Pathology, Case Western Reserve University, Cleveland, Ohio, USA; 19 University of Vic - Central University of Catalonia, Vic, Spain; 20 Institute of Family Medicine and Public Health, Tartu Ulikool Arstiteaduskond, Tartu, Estonia; 21 Department of Biostatistics, Johns Hopkins University, Baltimore, Maryland, USA; 22 Universitat Autònoma de Barcelona, Barcelona, Spain

**Keywords:** COVID-19, Epidemiology, Public Health, SARS-CoV-2

## Abstract

**Objectives:**

We aimed to assess the risk of incident autoimmune and inflammatory conditions during the post-acute period of COVID-19.

**Design:**

Descriptive network cohort study.

**Setting:**

Electronic health records from the UK and Dutch primary care, Norwegian linked health registry, hospital records of specialist centres in Spain, France and Korea and healthcare claims from Estonia and the USA.

**Participants:**

We followed individuals between September 2020 and the latest available data from day 91 after a SARS-CoV-2 negative test (comparator) or a COVID-19 record (exposed patients, ie assessing patients during the post-acute phase). We further established a reinfection cohort (any further COVID-19 record among the exposed patients). We followed patients until an outcome, end of study period, death, day 365 or an infection (comparator only) or reinfection (exposed patients only).

**Main outcome measures:**

We assessed postural orthostatic tachycardia syndrome (POTS) diagnoses/symptoms, myalgic encephalomyelitis/chronic fatigue syndrome (ME/CFS) diagnoses/symptoms, multi-inflammatory syndrome (MIS) and several autoimmune diseases (rheumatoid arthritis (RA), juvenile idiopathic arthritis (JIA), systemic lupus erythematosus (SLE), inflammatory bowel disease (IBD) and type 1 diabetes mellitus (T1DM)).

Meta-analysed crude incidence rate ratios (IRRs) of outcomes after COVID-19 versus negative testing and after reinfection versus a previous COVID-19 record yield the ratios of respective absolute risks of each assessed outcome. We performed subgroup analyses by age, sex and predominant variant periods.

**Results:**

We included 2 521 812 individuals with a first COVID-19 record, 4 233 145 with a first negative test and 135 551 with a reinfection. Age and sex were largely comparable between exposure groups with a shorter follow-up for the reinfection cohorts. After COVID-19 compared with test-negative patients and equally after reinfection compared with previous COVID-19 patients, we did not observe increased rates for all outcomes and all subgroup analyses. Counts of MIS and JIA were too small for meta-analyses.

**Conclusions:**

In our descriptive meta-analyses of crude IRRs among databases from various countries and settings, we did not observe increased rates of incident POTS, ME/CFS, RA, IBD, SLE and T1DM in COVID-19 versus test-negative or reinfection versus COVID-19 during the first 9 months of the post-acute phase of COVID-19 or reinfection (>90 days postinfection until month 12). Since causal interpretation cannot be made from this study, further causal research is warranted.

**WHAT IS ALREADY KNOWN ON THIS TOPIC**

- Basic and observational research suggested the existence of post-acute COVID-19 conditions, whose spectrum and timing are yet to be established.

**WHAT THIS STUDY ADDS**

- This study provides meta-analysed rate ratios of incident autoimmune and inflammatory conditions during the first 9 months of the post-acute phase of COVID-19 or reinfection, highlighting no increased ratios.

**HOW THIS STUDY MIGHT AFFECT RESEARCH, PRACTICE OR POLICY**

- Our findings from the early post-acute phase of COVID-19 shall instigate research with longer follow-ups as autoimmune and inflammatory conditions may take longer for diagnosing, especially during a pandemic and in the aftermath of COVID-19 when patients often present with multiple non-specific symptoms.

## Introduction

 The aftermath of COVID-19 continues to reveal a complex landscape of health challenges, with a significant subset of individuals experiencing prolonged symptoms beyond the acute phase of infection.^1,2^ Characterised by a diverse array of persistent symptoms encompassing fatigue, cognitive dysfunction and dysautonomia, it poses a challenge to both patients and healthcare providers.^3^ Worldwide estimates from meta-analysed data indicate that around one third of individuals may experience prolonged symptoms following acute COVID-19 and up to half following hospitalisation.^4,5^ The investigation of new-onset diagnoses emerging in the chronic phase, typically beyond 90 days postinfection, is clinically meaningful because it reduces misclassification from acute-phase complications and allows for the identification of incident conditions potentially triggered by long-term immune, autonomic or inflammatory dysregulation. On one hand, the close interplay between the immune system and the autonomic nervous system underscores the link between COVID-19 and conditions like postural orthostatic tachycardia syndrome (POTS).^6–12^ On the other hand, the precise link between COVID-19 and myalgic encephalomyelitis/chronic fatigue syndrome (ME/CFS) is still being elucidated. Emerging evidence suggests that COVID-19 may predispose some individuals to develop ME/CFS-like symptoms or exacerbate pre-existing ME/CFS.^12–15^ In the RECOVER-Adult cohort, 4.5% of infected adults developed new ME/CFS diagnoses within 6 months, compared with 0.6% in uninfected controls.^16^ Additionally, the emergence of multisystem inflammatory syndrome (MIS) in children (MIS-C) and adults (MIS-A) underscores the diverse inflammatory responses elicited by the virus.^17^ Finally, COVID-19 has been shown to trigger dysregulated immune responses, including the production of autoantibodies and the activation of inflammatory pathways, which may predispose individuals to the development or exacerbation of autoimmune conditions.^18^ Observational research suggests positive associations of COVID-19 with inflammatory disorders such as rheumatoid arthritis (RA), inflammatory bowel disease (IBD) or systemic lupus erythematosus (SLE) within various time frames.^19–21^

Elucidating the risk of POTS, ME/CFS, MIS and autoimmune diseases is paramount for understanding their impact on individuals, society and healthcare systems. Therefore, we aimed to assess the risks of these conditions in several countries and populations and provide meta-analysed results.

## Methods

### Study design

We performed a descriptive cohort study in an international network using routinely collected healthcare data mapped to the observational medical outcomes partnership common data model. Each site ran the study using a common analytical code and shared back results without sharing patient level data.^22–24^

### Data sources

Nine databases from six European countries, the USA and Korea contributed data to this study. Table 1 lists an overview of the characteristics of the individual databases, whether information on COVID-19 tests was available and decodes acronyms. Clinical Practice Research Datalink (CPRD) GOLD and CPRD Aurum contain primary care electronic health records (EHRs) registered with general practices (GPs) from an age and sex representative sample of 5% and 20% of inhabitants, respectively, mainly from Wales, Northern Ireland and Scotland (CPRD GOLD) and mainly from England (CPRD Aurum).^25–28^ Of note, data capture in CPRD GOLD occurs in practices with rather higher socioeconomic status than the general UK population while practices contributing to CPRD Aurum are located in areas representative of the socioeconomic distribution. The Integrated Primary Care Information (IPCI) contains EHRs collected from patients registered with GPs in the Netherlands.^29^ Norwegian Linked Health Registry data held by the University of Oslo (NLHR@UIO) data includes secondary care and prescription information from both primary and secondary care.^30^ IMASIS is an EHR hospital database containing records from patients being treated at the Hospital del Mar, Barcelona, Spain.^31^ eDOL CHUM (further referred to as CHUM) is an EHR hospital database containing records from patients being treated at the University Hospital Montpellier, France.^32^ CORIVA contains national health insurance claims from Estonia.^33^ AUSOM is an EHR hospital database containing records from patients being treated at Ajou University Medical Centre, Suwon, Republic of Korea.^34^ P+ contains medical and pharmacy claims from more than 107 million unique enrolees in largely commercial health plans.^35^

**Table 1:** Overview of participating databases

Database name	Acronym	Country	Data type	Healthcare setting	# people	Record end	COVID-19 negative test
Clinical Practice Research Datalink GOLD	CPRD GOLD	UK	EHR	Primary care	21M	June 2022	Yes
Clinical Practice Research Datalink Aurum	CPRD Aurum	UK	EHR	Primary care	40M	March 2022	Yes
The Integrated Primary Care Information	IPCI	The Netherlands	EHR	Primary care	3M	December 2022	Yes
Norwegian Linked Health Registry data held by the University of Oslo	NLHR@UIO	Norway	Administrative health-related data	Primary care and secondary care	5M	December 2021	No
Hospital records from Parc Salut Mar Barcelona	IMASIS	Spain	EHR	Secondary care	2M	December 2022	Yes
Hospital records from the Montpellier University Hospital	CHUM	France	EHR	Secondary care	2M	December 2022	Yes
Hospital records from Ajou University Medical Centre	AUSOM	Korea	EHR	Secondary care	2.7M	February 2023	Yes
Healthcare claims from Estonia	CORIVA	Estonia	Claims	Primary care and secondary care	300K	December 2022	Yes
PharMetrics Plus for Academics	P+	USA	Claims	Primary care and secondary care	107M	June 2022	No

EHR, electronic health records; M, million.

### Study population

**Figure 1:**
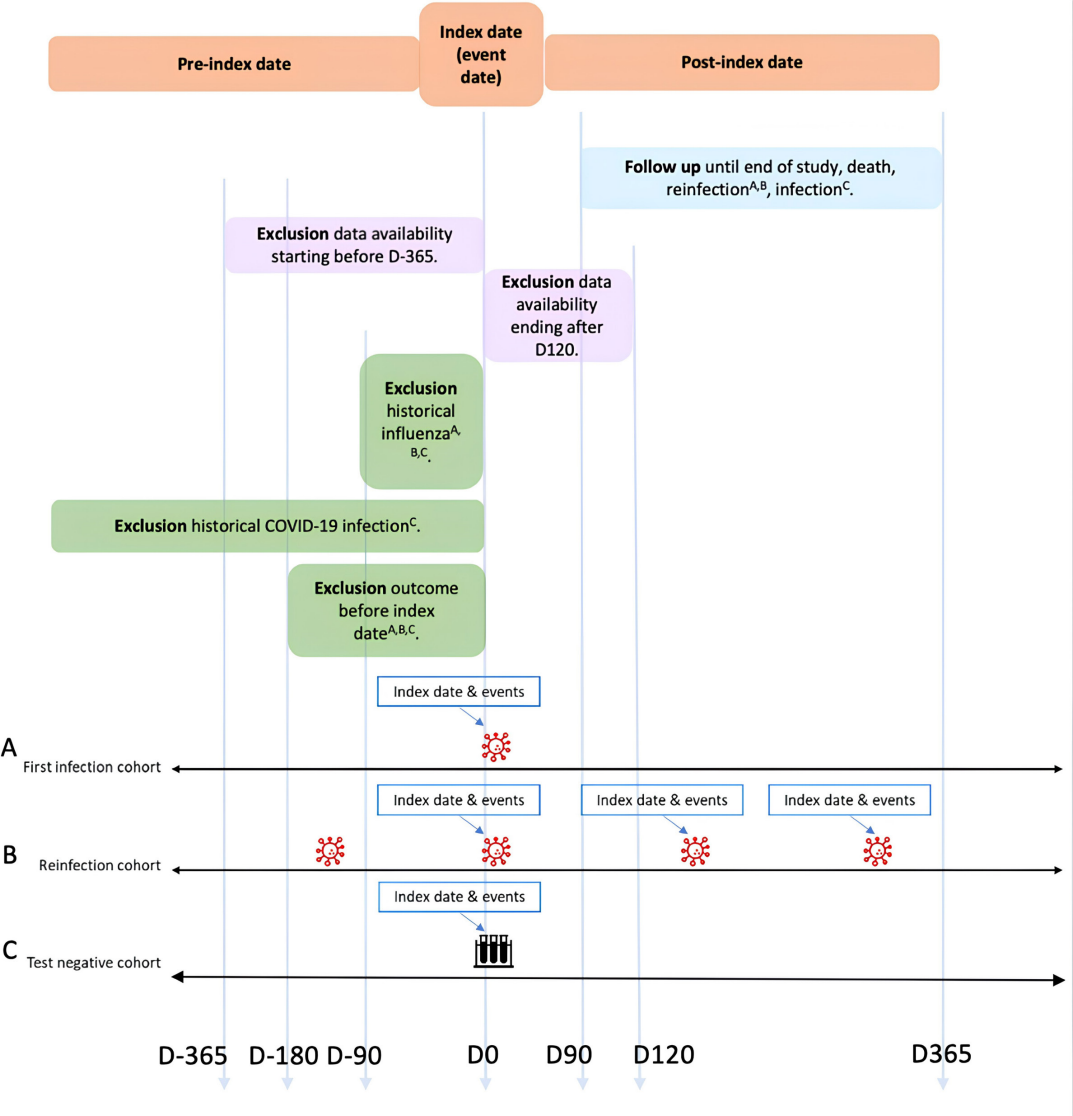
Graphical sketch of inclusion criteria for the three cohorts of the study. D, day.

The study period started on 1 September 2020 and continued until the latest data availability for each contributing database (table 1). The following events defined the index date for three corresponding cohorts: a first test-negative COVID-19 result for the test-negative comparator cohort, a first SARS-CoV-2 infection or clinical diagnosis for the COVID-19 cohort and any subsequent infection for the reinfection cohort (figure 1). All three cohorts shared key inclusion criteria: at least 365 days of prior observation before the index date, no influenza diagnosis within 90 days before the index date, no outcome of interest within 180 days before the index date (for each outcome analysis separately) and at least 120 days of potential follow-up after the index date (figure 1). Individual inclusion criteria included: for the test-negative cohort, to have a first recorded negative SARS-CoV-2 test during the study period, with no prior COVID-19 diagnosis or positive test (only one entry per person was allowed); for the COVID-19 cohort, to have a first recorded COVID-19 diagnosis (confirmed or suspected) or a positive SARS-CoV-2 test result (PCR or rapid lateral flow test) during the study period, with no prior COVID-19 diagnosis or positive test before the index date. Individuals were entered at the time of a second or subsequent COVID-19 record into the reinfection cohort. In this cohort, prior COVID-19 records were allowed, and individuals could contribute multiple episodes if they experienced multiple reinfections during the study period. Each reinfection episode had its own index date corresponding to the date of the subsequent COVID-19 diagnosis or positive test. Individuals could contribute at most once to the infection and test-negative cohorts but could contribute multiple episodes to the reinfection cohort. An individual initially included in the test-negative cohort who later tested positive could subsequently enter the infection or reinfection cohort if eligibility criteria were fulfilled. In such cases, follow-up in the previous cohort was censored at the time of infection to ensure no overlap. For all analytic cohorts, follow-up started on day 91 after the index date to exclude the acute phase of infection and continued until the earliest of outcome occurrence, end of data collection, death, day 365, COVID-19 (test-negative patients only), or reinfection (COVID-19 cohort only). Detailed code lists for exposures and exclusions are publicly available at https://github.com/oxford-pharmacoepi/LongCovidStudyathon_W1/tree/main/1_InitialCohorts/Jsons.

**Table 2:** Patient characteristics stratified by database and cohort

	Test-negative	COVID-19	Reinfection
CPRD GOLD			
# patients	669 155	352 178	8752
Female, n (%)	371 319 (55)	191 578 (54)	5456 (62)
Median age (IQR) (years)	38 (20–54)	35 (19–51)	30 (21–43)
Prior obs. (IQR) (years)	12 (5–18)	14 (7–18)	16 (8–19)
Median time in cohort (days)	298	241	159
CPRD AURUM			
# patients	3 297 889	1 023 461	10 241
Female, n (%)	1 782 808 (54)	538 899 (53)	6077 (59)
Median age (IQR) (years)	35 (18–52)	33 (17–49)	31 (18–47)
Prior obs. (IQR) (years)	11 (5–19)	11 (5–19)	203 (157–248)
Median time in cohort (days)	345	250	205
IPCI			
# patients	1459	181 815	11 619
Female, n (%)	895 (61)	97 711 (54)	6943 (59)
Median age (IQR) (years)	58 (39–74)	39 (21–55)	36 (22–50)
Prior obs. (IQR) (years)	6 (4–7)	7 (3–10)	8 (5–10)
Median time in cohort (days)	350	339	324
NLHR@UIO			
# patients	NA	698 538	97 073
Female, n (%)	NA	349 625 (50)	53 948 (54)
Median age (IQR) (years)	NA	33 (19–50)	31 (20–45)
Prior obs. (IQR) (years)	NA	6 (3–10)	207 (147–260)
Median time in cohort (days)	NA	335	207
IMASIS			
# patients	27 703	13 939	1713
Female, n (%)	15 088 (54)	7300 (52)	918 (49)
Median age (IQR) (years)	60 (41–75)	58 (42–72)	62 (50–73)
Prior obs. (IQR) (years)	17 (8–26)	16 (8–26)	18 (10–28)
Median time in cohort (days)	233	231	189
CHUM			
# patients	73 187	6121	88
Female, n (%)	40 668 (56)	3444 (56)	44 (48)
Median age (IQR) (years)	43 (28–66)	49 (30–71)	50 (34.5–68.5)
Prior obs. (IQR) (years)	31 (29–32)	31 (31–32)	32 (32–32)
Median time in cohort (days)	362	364	310
AUSOM			
# patients	30 065	598	NA
Female, n (%)	15 953 (53)	321 (54)	NA
Median age (IQR) (years)	55 (38–68)	50 (29–68)	NA
Prior obs. (IQR) (years)	11 (5–19)	12 (5–20)	NA
Median time in cohort (days)	264	253	NA
CORIVA			
# patients	133 687	43 215	2282
Female, n (%)	71 422 (53)	23 351 (54)	1373 (59)
Median age (IQR) (years)	39 (25–55)	35 (20–51)	35 (20–48)
Prior obs. (IQR) (years)	4 (4–4)	4 (4–4)	5 (4–5)
Median time in cohort (days)	344	348	194
P+			
# patients	NA	201 947	3783
Female, n (%)	NA	105 983 (52)	2115 (55)
Median age (IQR) (years)	NA	43 (27–59)	49 (30–64)
Prior obs. (IQR) (years)	NA	4 (2–4)	4 (2–5)
Median time in cohort (days)	NA	330	246

AUSOM, Hospital records from Ajou University Medical Centre; CHUM, Hospital records from the Montpellier University Hospital; CORIVA, Healthcare claims from Estonia; CPRD AURUM, Clinical Practice Research Datalink AURUM; CPRD GOLD, Clinical Practice Research Datalink GOLD; IMASIS, Hospital records from Parc Salut Mar Barcelona; IPCI, The Integrated Primary Care Information; NA, not available; NLHR@UIO, Norwegian Linked Health Registry data held by the University of Oslo; obs., observation; P+, PharMetrics Plus for Academics.

### Outcomes

Our outcomes were a first record of POTS diagnoses and POTS symptoms such as palpitations, sinus tachycardia, vertigo, sweating, syncope, lightheadedness, orthostatic hypotension, dyspnoea, fatigue, dizziness and giddiness (code list ‘dysautonomia’). Moreover, we assessed a first record of ME/CFS diagnoses and ME/CFS symptoms such as fatigue, malaise, tiredness, irritable bowel syndrome, asthenia, dysthymia, heavy feeling and heavy legs. The symptoms code lists were very sensitive and comprised any symptoms largely related to the disease to capture any potential diagnoses of POTS or ME/CFS not coded as such due to the uncertainty around diagnosing these diseases. Furthermore, we assessed a first record of MIS and the following autoimmune diseases: RA, juvenile idiopathic arthritis (JIA), SLE, IBD, type 1 diabetes mellitus (T1DM). The absence of a recorded symptom/diagnosis was interpreted as absence of that symptom/diagnosis. Outcome code lists were established by TB and reviewed by DPA. Outcome code lists (SNOMED) are publicly available at https://github.com/oxford-pharmacoepi/LongCovidStudyathon_W1/tree/main/1_InitialCohorts/Jsons.

### Covariates

We performed stratified analyses by age (0–18 years, 19–64 years, >64 years), sex and predominant SARS-CoV-2 variant periods: Alpha (December 2020–June 2021), Delta (July 2021–14 December 2021), Omicron BA.1 (15 December 2021–31 March 2022) and Omicron BA.2+ (April 2022 onward).^36,37^ Variants were spreading across the globe with little delay which is why we used the same periods for all databases. Records of age and sex used for stratification were complete, with no missingness.

### Statistical analyses

Because of the many databases and different populations in this study, we only reported frequency and percentage of the sexes, median and IQR for age, prior observation and follow-up as baseline characteristics.

Following COVID-19 versus the test-negative cohort and following reinfection versus a previous COVID-19 record, we estimated crude incidence rate ratios (IRRs) with 95% CIs for all outcomes individually.^38^ We further conducted a random effects meta-analysis using the restricted maximum-likelihood estimator for the between-study variance (τ²) and assessed heterogeneity using the I² statistic based on the Q statistic. These parameters allowed us to account for differences across databases and quantify the extent of between-database variability. We assessed heterogeneity and further performed sensitivity analyses using Hartung-Knapp (HK) adjustments when heterogeneity was high. We explored subgroup analyses by age, sex and SARS-CoV-2 variant (approximated by the predominant temporal periods).

As a sensitivity analysis to depict IRR adjusted for age and sex, we additionally fitted Poisson regression models within each database including age and sex as covariates, followed by pooling of the adjusted IRRs via random-effects meta-analysis. These Poisson regression models were estimated using a robust sandwich estimator to address potential cluster correlations resulting from patients entering multiple cohorts.

Where frequency counts were less than five, data were obscured to further enhance patient/practice confidentiality, that is, we have not reported IRR for these outcomes/strata.

All analyses were conducted using R V.4.2.3 (R Core Team, 2023), primarily relying on the packages PatientProfiles^39^ (to describe patient characteristics), IncidencePrevalence^40^ (to estimate disease incidence) and meta^38^ (to perform meta-analyses). The analytical code is publicly available at oxford-pharmacoepi/LongCovidStudyathon_W1 (github.com) and for meta-analyses and figures at immune_inflammatory_PACS/ at main · tiozab/immune_inflammatory_PACS (github.com).

### Patient and public involvement

We engaged with the public and presented the study to a patient group at the Nuffield Department of Orthopaedics, Rheumatology and Musculoskeletal Sciences (NDORMS) at the University of Oxford, UK. Furthermore, we discussed the study setup with a patient representative of the European Alliance of Associations for Rheumatology (EULAR).

## Results

Taking all the databases, we included a total of 2 521 812 individuals with COVID-19, 4 233 145 with a first negative test and 135 551 with a reinfection. CPRD Aurum was the biggest database with a total of 1 023 461 COVID-19 patients and 3 297 889 test-negative patients. Table 2 depicts patient characteristics of all databases. Cohorts were well balanced regarding age and sex except for IPCI which had a much older female COVID-19 cohort (but not for reinfection). Follow-up was comparable for all cohorts except reinfection cohorts.

**Figure 2:**
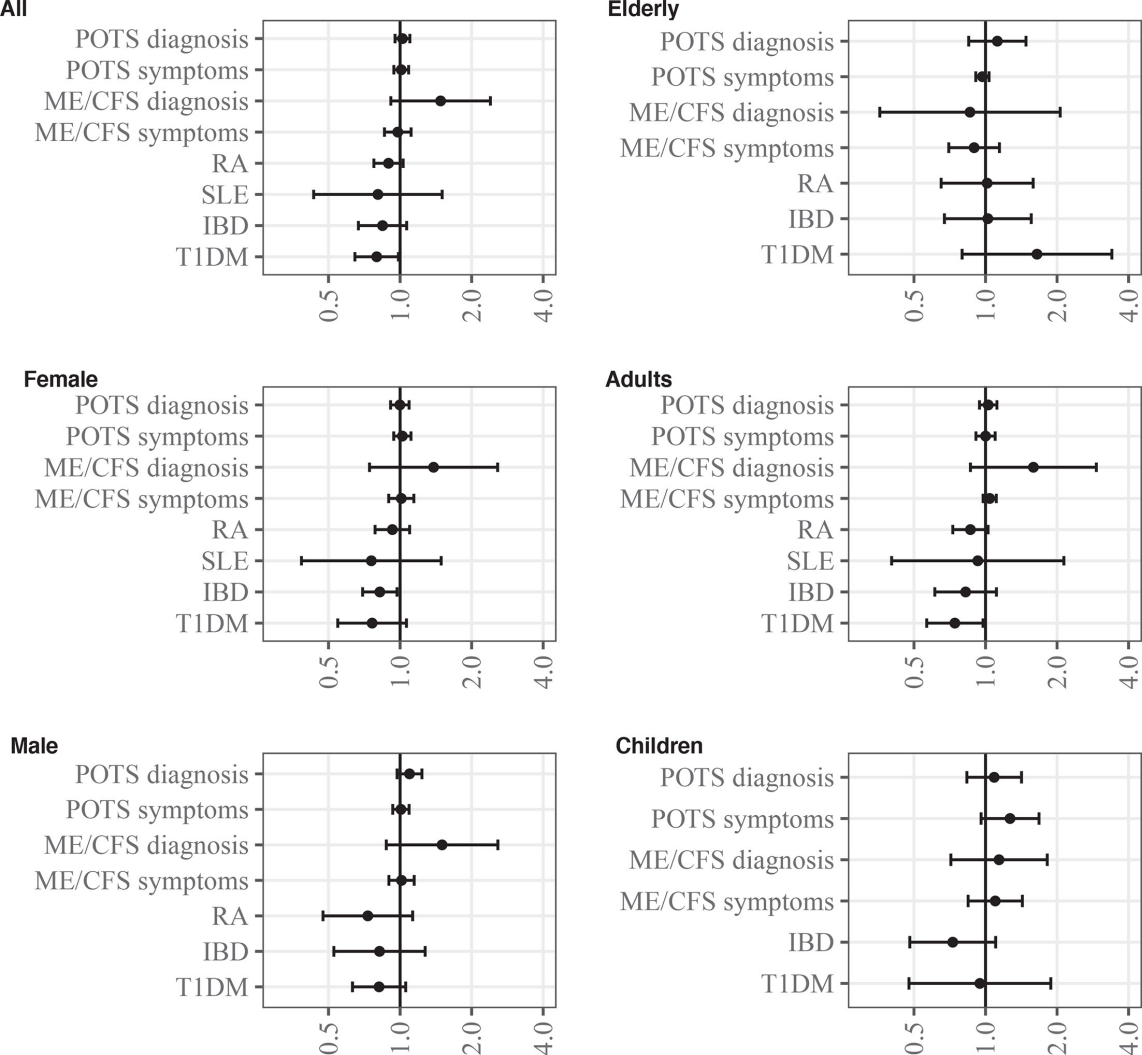
Meta-analysed crude incidence rate ratios with 95% CIs for all outcomes among COVID-19 versus test-negative patients, overall and stratified by age and sex. IBD, inflammatory bowel disease; ME/CFS, myalgic encephalomyelitis/chronic fatigue syndrome; POTS, postural orthostatic tachycardia syndrome; RA, rheumatoid arthritis; SLE, systemic lupus erythematosus; T1DM, type 1 diabetes mellitus.

Meta-analysed crude overall IRR after COVID-19 compared with after negative testing depicted IRRs of 1.02 (0.95 to 1.10), 1.01 (0.94 to 1.09), 0.98 (0.86 to 1.11), 1.48 (0.91 to 2.40), for POTS diagnoses, POTS symptoms, ME/CFS diagnoses and ME/CFS symptoms, respectively (figure 2, numeric values in online supplemental table 1). Results remained unchanged in subgroups of age and sex. For autoimmune diseases, we observed overall IRRs of 0.84 (0.67 to 1.07), 0.89 (0.78 to 1.03), 0.81 (0.43 to 1.50) and 0.80 (0.65 to 0.98), for IBD, RA, SLE and T1DM, respectively. We observed reduced rates of T1DM in adults with IRRs of 0.74 (0.57 to 0.98) and for IBD of 0.82 (0.7 to 0.97) among women only. JIA and MIS yielded too few counts for meta-analysed IRR analyses. Input data for the meta-analyses of IRRs are depicted in online supplemental table 2. Results per database are presented in online supplemental figures 1–6 and corresponding numeric values are presented in online supplemental table 3. Not all databases had sufficient counts for individual analyses, especially AUSOM had too few counts for all outcomes. Heterogeneity between databases per outcome is depicted in online supplemental figure 7. It was low for POTS diagnosis, RA, IBD and T1DM, that is, different databases depicted similar results and was moderate for IBD and POTS symptoms. ME/CFS symptoms and diagnoses yielded high heterogeneity and depicted different results even within the same country and healthcare setting, for example, ME/CFS diagnoses in CPRD GOLD and CPRD Aurum had IRRs of 2.03 (1.50 to 2.74) and 0.89 (0.74 to 1.07), respectively (online supplemental figures 3 and 4, numeric values in online supplemental table 3). Overall results for ME/CFS diagnoses and symptoms when using HK adjustments for high heterogeneity depicted slightly wider CIs than in the original analyses and remained null results (online supplemental table 4). Sensitivity analysis estimating Poisson age and sex adjusted IRRs were largely consistent with Poisson crude IRRs, and their CIs were overall similar in width (online supplemental figure 8). Compared with our original analysis, results were similar except for SLE which depicted decreased risks for all Poisson analyses while T1DM had a null result.

**Figure 3:**
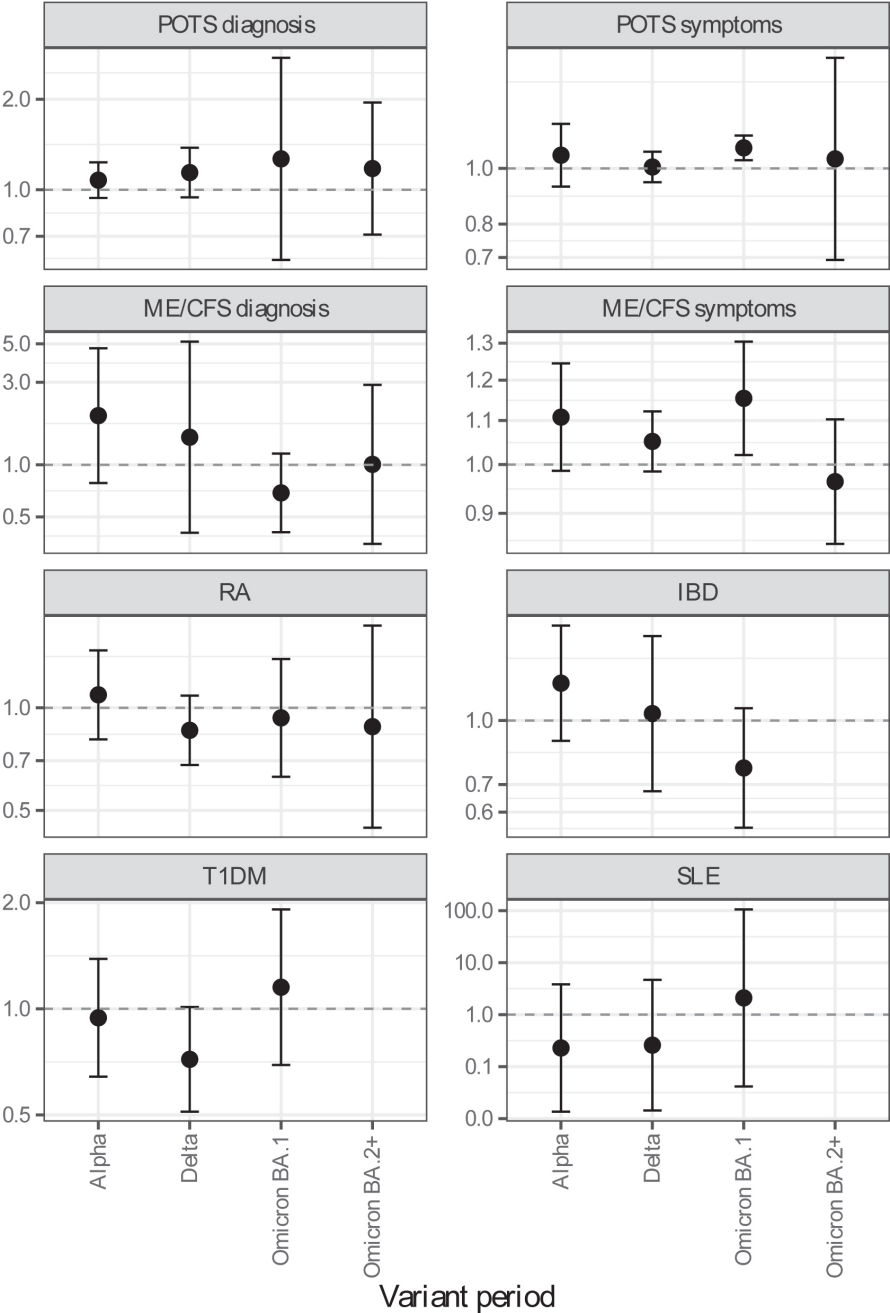
Meta-analysed crude incidence rate ratios with 95% CIs for all outcomes among COVID-19 versus test-negative patients overall stratified by predominant periods of individual variants. IBD, inflammatory bowel disease; ME/CFS, myalgic encephalomyelitis/chronic fatigue syndrome; POTS, postural orthostatic tachycardia syndrome; RA, rheumatoid arthritis; SLE, systemic lupus erythematosus; T1DM, type 1 diabetes mellitus.

**Figure 4:**
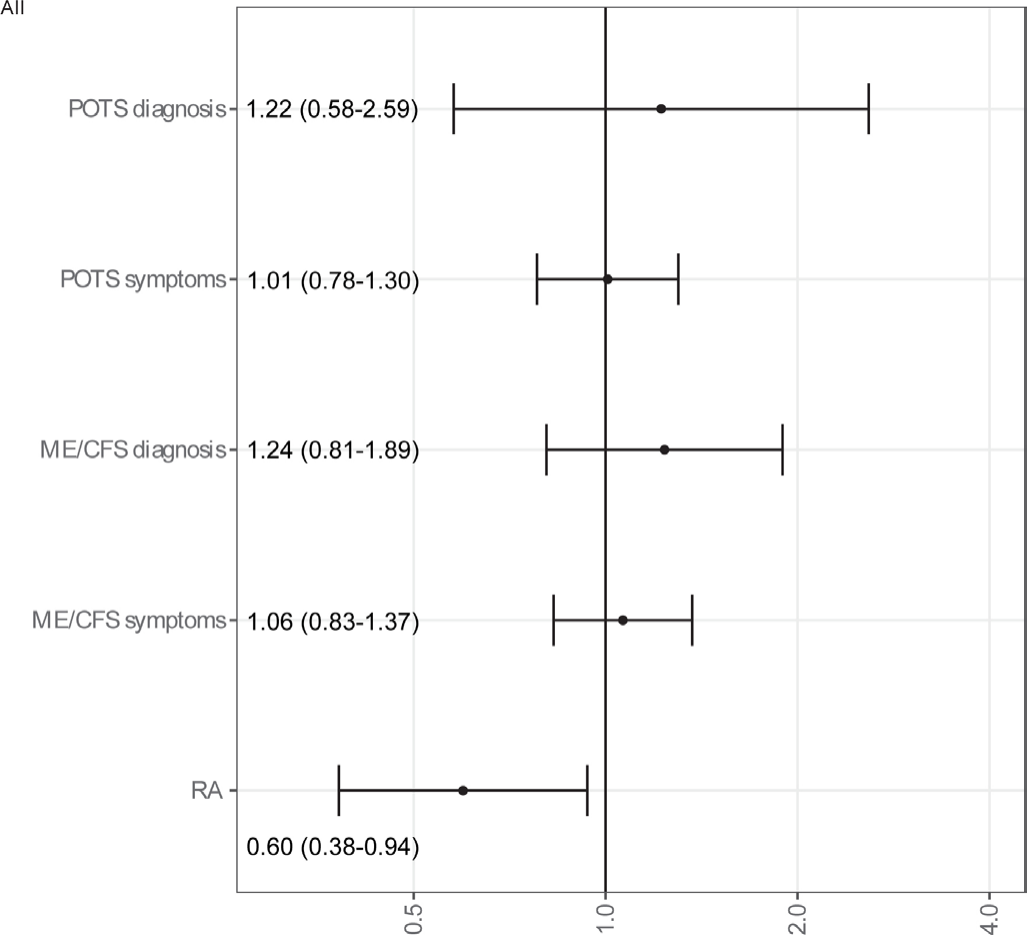
Meta-analysed crude incidence rate ratios with 95% CIs for all outcomes among reinfection versus COVID-19 overall. ME/CFS, myalgic encephalomyelitis/chronic fatigue syndrome; POTS, postural orthostatic tachycardia syndrome; RA, rheumatoid arthritis.

Analyses during the time periods of SARS-CoV-2 variants of interest yielded significant results for the Omicron BA.1 period for ME/CFS symptoms and POTS symptoms with IRRs of 1.09 (1.03 to 1.14) and 1.15 (1.02 to 1.30), respectively (figure 3, numeric values in online supplemental table 5). However, diagnoses of ME/CFS and POTS yielded null results. Finally, our meta-analyses of risks after reinfection versus a first COVID-19 record are depicted in figure 4 (numeric values in online supplemental table 6) and only had sufficient counts for the overall population assessing POTS diagnosis, POTS symptoms, ME/CFS diagnosis and ME/CFS symptoms and RA. We observed null results throughout except for RA with an IRR of 0.60 (0.38 to 0.94) whose result stemmed from one database only though (NLHR@UIO). Heterogeneity between databases per outcome is depicted in online supplemental figure 9 and was higher than in the COVID-19 versus test-negative cohort results (likely due to smaller outcome numbers).

## Discussion

In this large network study from nine databases in Europe, the USA and Korea, of whom ~3 million patients had COVID-19, ~4 million patients a negative test and >100 000 patients a reinfection, in meta-analyses, we did not observe increased rates for all assessed outcomes (POTS, ME/CFS, RA, IBD, SLE, T1DM) following COVID-19 compared with test-negative cohorts and reinfection compared with previous COVID-19. We assessed incident disease during the early post-acute phase (>90 days following infection and until month 12 thereafter) and did not observe substantial change in rates given age, sex or COVID-19 variant’s time period. Numbers for JIA and MIS were too small for analyses.

Our result suggesting that the rates of POTS after COVID-19 compared with after negative testing are not increased seems to differ from the general tone of the literature which mainly consists of the reporting of positive associations. Yet, a closer look into the timings of the published literature suggests that increased risks are observed during the acute COVID-19 phase. A systematic review and meta-analysis observed a twofold increased rate of POTS in infected versus uninfected.^41^ While this meta-analysis had a different comparator, it also consisted of only three studies out of which two studies had only one and two events (of note, these types of results were suppressed in our analyses due to privacy restrictions). The only major observational study with a significant number of patients (n=12 460) studied merely the first 3 months postinfection which we did not consider as part of the post-acute phase.^7^ Thus, our results add to current literature that incident POTS may not remain an issue for COVID-19 patients as of the post-acute period >90 days post-infection.

While we observed a slight and transient increase in rates of 1.09 and 1.15 of symptoms of POTS and ME/CFS, respectively, during the Omicron BA.1 period, this seemed to be a chance finding potentially due to altered and heterogeneous recording of these symptoms following the first positive case series publications. The increased finding does not persist for the other Omicron variant BA.2+ and, among all outcomes assessed, ME/CFS presented as the most heterogeneous between databases. Moreover, a study from Japan finds the lowest prevalence of ME/CFS during the Omicron period among all variant’s periods.^42^ Overall and for all subgroups of age and sex, we observed no increased ratios for ME/CFS. Our finding is consistent with a recent mendelian randomisation study suggesting no causal association between COVID-19 and ME/CFS.^43^ A meta-analysis on fatigue outcomes following COVID-19 suggests increased rates but also observes a time effect that suggests a reduction in this increase with time.^44^ Thus, our results add to this growing evidence base that incident ME/CFS may not remain an issue for COVID-19 patients as of the post-acute period >90 days postinfection.

Regarding the assessed autoimmune diseases, we observed various slightly decreased rates after COVID-19 or reinfection, yet none were consistent between analyses which suggests that these findings may be chance findings. Our findings of no increased rates contrast with existing literature mainly publishing increased risks of RA, IBD and other autoimmune diseases.^19–21^ However, published literature includes studies which assessed outcomes after 30 days from COVID-19 which seems too short to diagnose new autoimmune disease, thus including prevalent disease,^20^ and such that only had 6 months of follow-up postinfection which may be again too short to diagnose autoimmune diseases.^21^ Our follow-up only started at month 4 decreasing the chances of detection of pre-existing conditions and was slightly longer (until month 12). A study assessing incident RA in Columbia depicts that prevalence of seropositive RA in their database dropped from 87% in 2020 to 46% in 2022 while unspecified and other RA-related conditions saw an increase from 11% to 39% and 0% to 10%, respectively. This shift in records showcases the uncertainty around postinfection autoimmune disease diagnosing during the COVID-19 pandemic when patients often presented with non-specific symptoms. Furthermore, additional reports of glucometabolic control failing during and after COVID-19 potentially being recorded as T1DM (potentially because of insulin records) may not reflect true T1DM but just a transient symptom.^45,46^ By focusing on first-time diagnoses ≥90 days post-infection, our study mitigated misclassification of short-term inflammation and ensured incident T1DM cases reflected true post-acute phase cases. Moreover, it is important to recognise that autoimmune diagnosis pathways were disrupted during the COVID-19 pandemic. A regional Italian study reported an around 30% decrease in testing and first visits, though a stable rate of positive diagnoses—particularly for severe rheumatic diseases, which may indicate delays for milder conditions.^47^ Thus, our observed decreased rates after COVID-19 might be due to symptoms of autoimmune disease being non-specific or mild and being handled as post-acute COVID-19 symptoms, rather than a separate disease, hence leading to delayed diagnoses.

Inflammatory and autoimmune disease take time to diagnose, and for RA and POTS, for example, this has been reported to take around 2 years.^48,49^ Since our study only assessed follow-up until month 12, we likely only captured severe or clear cases, and we suggest that further long-term studies are conducted.

Our study has the strengths of producing a large quantity of standardised results from various countries and care settings in Europe. Our meta-analysed results provide reliable evidence and reduce the chance of reporting spurious findings as had been observed in individual databases for ME/CFS in this study, for example, and may result in publication bias. Trends for other outcomes were largely similar among the individual databases which underlines the robustness of our null findings. Moreover, we used test-negative comparators which are more reliable than comparing to patients without COVID-19 because they may have still been infected at the time. This choice of comparator also controls for confounding by healthcare seeking behaviour.

However, we must consider several limitations, some inherent to using electronic healthcare data in this situation, some inherent to the chosen study design. First, assessing relatively unknown diseases such as POTS and ME/CFS which suddenly came to the spotlight may have resulted in different reporting or coding by medical professionals in different healthcare settings and countries. Thus, given the lack of ubiquitous diagnostic criteria there is an increased risk of outcome misclassification. Related underlying time trends given emerging literature concerning post-COVID-19 conditions may have further influenced reporting habits and skewed analyses of emerging variants over time. Additionally, results of POTS and ME/CFS symptoms from more sensitive code lists may also be subject to bias from different usage of codes for symptoms which may not be considered as important as codes for diseases in some instances by different healthcare professionals and settings.

Second, since investigated diseases may take a long time to be diagnosed, and are in essence rule-out diagnoses (diagnoses that may later be changed to another disease), their incidence rates may be artificially low and potentially differently so between groups resulting in biased results. Reinfection cohorts had the shortest observation time and were therefore most prone to a potentially insufficient follow-up time for a recorded outcome. Moreover, while we used a 3-month run-in period in which we did not assess outcomes, we may have still captured patients with pre-existing conditions not yet recorded as such which are not true incident cases and may have biased our results.

Third, misclassification of exposure was possible. While a first COVID-19 and test-negative test were considered to be well recorded, reinfection with COVID-19 was less so because often less severe. While biasing our results, it also limits the representativeness since individuals with documented reinfections may have been rather healthcare seeking individuals, more severe cases, or patients in settings where testing was more prominent. Furthermore, for variant-adjusted analyses, infection variant was assigned according to the dominant variant at the infection date and represents therefore only an approximation of the actual variant with possible misclassifications, especially at the boundaries of the time frames.

Fourth, we did not match our comparison groups and did not adjust for confounders such as comorbidities, COVID-19 vaccination, lifestyle or socioeconomic factors. Yet, we found age and sex mostly balanced in our cohorts and adjusted IRR from Poisson models were largely consistent with our original analyses. Nonetheless, residual confounding is likely: individuals with different health-seeking behaviour, pre-existing comorbidities, access to healthcare, vaccination status may be distributed unevenly between, potentially biasing our results in either direction. Differences in referral pathways and access to specialist diagnostic services across settings and countries may have further influenced our results.

Fifth, we did not include outcomes not causally related to the exposure (ie, negative control outcomes) in our study which would have helped to further validate our findings or detect bias and confounding.

Finally, given the descriptive nature of our analyses reporting comparative occurrence in recorded healthcare activity, the results should not be interpreted as causal.

## Conclusions

In our descriptive meta-analyses of crude IRRs from databases from various continents and healthcare settings, we observed that, as of the post-acute phase of COVID-19 or reinfection, incident POTS, ME/CFS, RA, IBD, SLE and T1DM did not yield higher rates than after negative testing or a previous COVID-19 record, respectively. Our follow-up ends at month 12 postinfection and may not allow sufficient time for diagnosing inflammatory and autoimmune diseases, thus further studies with long-term follow-up are needed. Since causal interpretation cannot be made from this study, further causal research is warranted.

## Supplementary material

10.1136/bmjph-2024-001686 online supplemental figure 1

10.1136/bmjph-2024-001686 online supplemental figure 2

10.1136/bmjph-2024-001686 online supplemental figure 3

10.1136/bmjph-2024-001686 online supplemental figure 4

10.1136/bmjph-2024-001686 online supplemental figure 5

10.1136/bmjph-2024-001686 online supplemental figure 6

10.1136/bmjph-2024-001686 online supplemental figure 7

10.1136/bmjph-2024-001686 online supplemental figure 8

10.1136/bmjph-2024-001686 online supplemental figure 9

10.1136/bmjph-2024-001686 online supplemental table 1

10.1136/bmjph-2024-001686 online supplemental table 2

10.1136/bmjph-2024-001686 online supplemental table 3

10.1136/bmjph-2024-001686 online supplemental table 4

10.1136/bmjph-2024-001686 online supplemental table 5

10.1136/bmjph-2024-001686 online supplemental table 6

## Data Availability

Data may be obtained from a third party and are not publicly available.
