# Supplementary material for: Risks of autoimmune and inflammatory post-acute COVID-19 conditions: a network cohort study in six European countries, the USA and Korea
Source: BMJ Public Health. 2026 Jul 24;4(3):e001686. doi: 10.1136/bmjph-2024-001686 (PMC13404851; doi:10.1136/bmjph-2024-001686)
Supplement: online supplemental figure 2 [file bmjph-4-3-s002.docx]

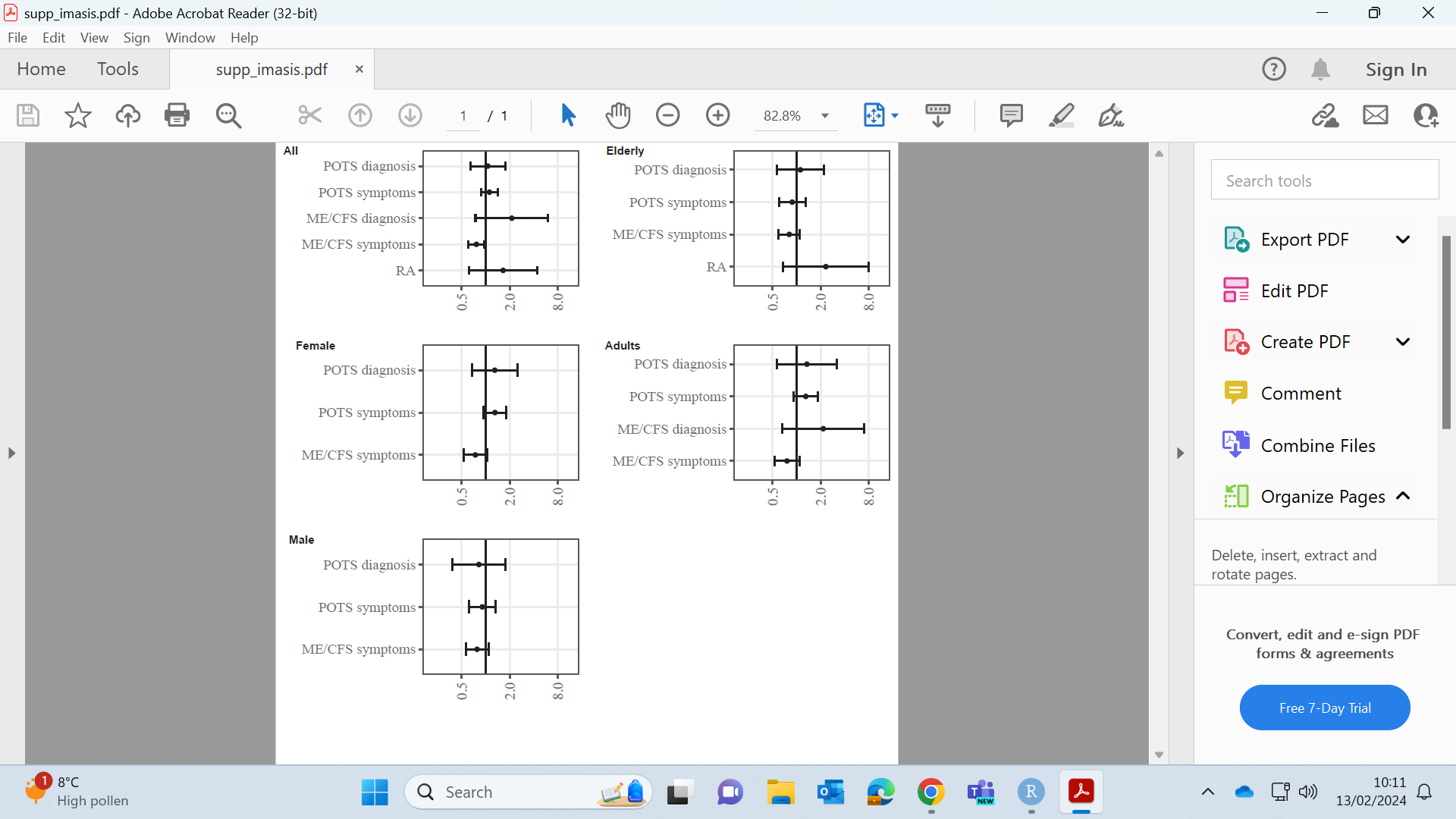


#### Supplementary Figure 2. Incidence rate ratio results in IMASIS database.

Numeric values available in Supplementary Table 2

ME/CFS: myalgic encephalomyelitis / chronic fatigues syndrome; POTS: postural orthostatic tachycardia syndrome; RA: rheumatoid arthritis;
