# Supplementary material for: Risks of autoimmune and inflammatory post-acute COVID-19 conditions: a network cohort study in six European countries, the USA and Korea
Source: BMJ Public Health. 2026 Jul 24;4(3):e001686. doi: 10.1136/bmjph-2024-001686 (PMC13404851; doi:10.1136/bmjph-2024-001686)
Supplement: online supplemental figure 8 [file bmjph-4-3-s008.docx]

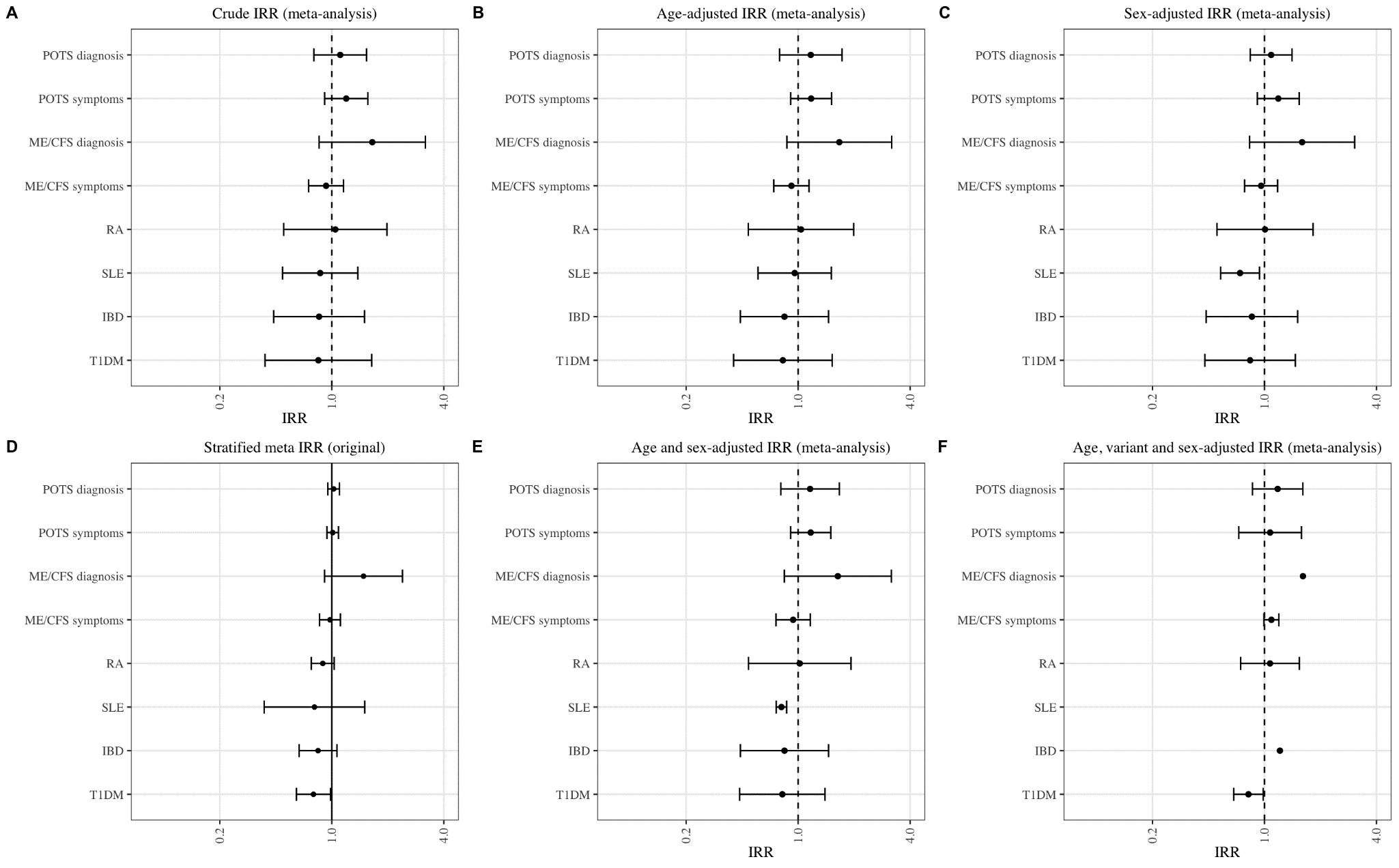


*Supplementary Figure 8. Poisson fitted meta-analysed IRR*

A: crude Poisson model; B: age adjusted Poisson model; C: sex adjusted Poisson model; D: original analysis (for comparison); E: age- and sex adjusted Poisson model; F: age-, virus variant- and sex adjusted Poisson model (ME/CFS diagnosis and IBD had confidence intervals that went beyond the axes and they are therefore not depicted in the graph, SLE had <5 outcomes at this stratification level for each strata and therefore has no results)

IBD: inflammatory bowel disease; ME/CFS: myalgic encephalomyelitis / chronic fatigues syndrome; POTS: postural orthostatic tachycardia syndrome; RA: rheumatoid arthritis; SLE: systemic lupus erythematosus; T1DM: type 1 diabetes mellitus
