# Supplementary material for: Risks of autoimmune and inflammatory post-acute COVID-19 conditions: a network cohort study in six European countries, the USA and Korea
Source: BMJ Public Health. 2026 Jul 24;4(3):e001686. doi: 10.1136/bmjph-2024-001686 (PMC13404851; doi:10.1136/bmjph-2024-001686)
Supplement: online supplemental table 1 [file bmjph-4-3-s010.docx]

*Supplementary Table 1. Numeric values of incidence rate ratios with 95% confidence intervals corresponding to Figure 2*

|  | **All** | **Female** | **Male** | **Elderly**  **(>64 years** | **Adults**  **(19-64 years)** | **Children**  **(<19 years)** |
| --- | --- | --- | --- | --- | --- | --- |
| **POTS diagnosis** | 1.02 (0.95-1.10) | 1.00 (0.91-1.09) | 1.1 (0.97-1.24) | 1.12 (0.85-1.48) | 1.03 (0.94-1.12) | 1.09 (0.83-1.42) |
| **POTS symptoms** | 1.01 (0.94-1.09) | 1.02 (0.94-1.11) | 1.01 (0.93-1.09) | 0.97 (0.91-1.03) | 1.00 (0.91-1.1) | 1.27 (0.96-1.68) |
| **ME/CFS diagnosis** | 1.48 (0.91-2.40) | 1.38 (0.74-2.57) | 1.50 (0.87-2.58) | 0.86 (0.36-2.06) | 1.59 (0.86-2.93) | 1.14 (0.71-1.82) |
| **ME/CFS symptoms** | 0.98 (0.86-1.11) | 1.01 (0.90-1.14) | 1.01 (0.90-1.15) | 0.90 (0.70-1.14) | 1.04 (0.98-1.11) | 1.10 (0.84-1.43) |
| **RA** | 0.89 (0.78-1.03) | 0.93 (0.78-1.1) | 0.73 (0.47-1.13) | NA | 0.86 (0.73-1.02) | 4.22 (0.08-212.81) |
| **IBD** | 0.84 (0.67-1.07) | 0.82 (0.70-0.97) | 0.82 (0.53-1.27) | 1.02 (0.67-1.56) | 0.82 (0.61-1.11) | 0.73 (0.48-1.10) |
| **SLE** | 0.81 (0.43-1.50) | 0.76 (0.38-1.49) | NA | NA | 0.93 (0.40-2.14) | NA |
| **T1DM** | 0.80 (0.65-0.98) | 0.76 (0.55-1.06) | 0.82 (0.63-1.06) | 1.65 (0.8-3.4) | 0.74 (0.57-0.98) | 0.95 (0.48-1.88) |

IBD: inflammatory bowel disease; ME/CFS: myalgic encephalomyelitis / chronic fatigues syndrome; NA: results suppressed because less than 5 outcomes; POTS: postural orthostatic tachycardia syndrome; RA: rheumatoid arthritis; SLE: systemic lupus erythematosus; T1DM: type 1 diabetes mellitus
