# Supplementary material for: Risks of autoimmune and inflammatory post-acute COVID-19 conditions: a network cohort study in six European countries, the USA and Korea
Source: BMJ Public Health. 2026 Jul 24;4(3):e001686. doi: 10.1136/bmjph-2024-001686 (PMC13404851; doi:10.1136/bmjph-2024-001686)
Supplement: online supplemental table 4 [file bmjph-4-3-s013.docx]

*Supplementary Table 4. Incidence rate ratios with 95% confidence intervals of outcomes with high heterogeneity*

|  | **All** | **Female** | **Male** | **Elderly**  **(>64 years** | **Adults**  **(19-64 years)** | **Children**  **(<19 years)** |
| --- | --- | --- | --- | --- | --- | --- |
| **ME/CFS diagnosis** | 1.02 (0.93-1.12) | 1.00 (0.88-1.13) | 1.10 (0.92-1.30) | 1.12 (0.75-1.68) | 1.03 (0.91-1.16) | 1.09 (0.81-1.46) |
| **ME/CFS symptoms** | 1.01 (0.93-1.10) | 1.02 (0.91-1.15) | 1.01 (0.93-1.10) | 0.97 (0.93-1.01) | 1.00 (0.87-1.16) | 1.27 (0.65-2.48) |

ME/CFS: myalgic encephalomyelitis / chronic fatigues syndrome; NA: results suppressed because less than 5 outcomes;
