# Supplementary material for: Risks of autoimmune and inflammatory post-acute COVID-19 conditions: a network cohort study in six European countries, the USA and Korea
Source: BMJ Public Health. 2026 Jul 24;4(3):e001686. doi: 10.1136/bmjph-2024-001686 (PMC13404851; doi:10.1136/bmjph-2024-001686)
Supplement: online supplemental table 5 [file bmjph-4-3-s014.docx]

*Supplementary Table 5. Numeric values of incidence rate ratios with 95% confidence intervals corresponding to Figure 3*

|  | **Alpha variant** | **Delta variant** | **Omicron BA.1 variant** | **Omicron BA.2+** **variant** |
| --- | --- | --- | --- | --- |
| **POTS diagnosis** | 1.08 (0.94-1.23) | 1.14 (0.94-1.38) | 1.27 (0.58-2.75) | 1.18 (0.71-1.95) |
| **POTS symptoms** | 1.05 (0.93-1.20) | 1.01 (0.95-1.07) | 1.09 (1.03-1.14) | 1.04 (0.69-1.56) |
| **ME/CFS diagnosis** | 1.92 (0.78-4.72) | 1.44 (0.40-5.15) | 0.69 (0.41-1.16) | 1.01 (0.35-2.90) |
| **ME/CFS symptoms** | 1.11 (0.99-1.24) | 1.05 (0.99-1.12) | 1.15 (1.02-1.30) | 0.96 (0.84-1.10) |
| **RA** | 1.09 (0.81-1.47) | 0.86 (0.68-1.09) | 0.93 (0.63-1.39) | 0.88 (0.44-1.74) |
| **IBD** | 1.23 (0.89-1.70) | 1.04 (0.67-1.60) | 0.77 (0.55-1.07) | NA |
| **SLE** | 0.23 (0.01-3.83) | 0.26 (0.01-4.68) | 2.1 (0.04-105.6) | NA |
| **T1DM** | 0.94 (0.64-1.39) | 0.72 (0.51-1.01) | 1.15 (0.69-1.91) | NA |

IBD: inflammatory bowel disease; ME/CFS: myalgic encephalomyelitis / chronic fatigues syndrome; NA: results suppressed because less than 5 outcomes; POTS: postural orthostatic tachycardia syndrome; RA: rheumatoid arthritis; SLE: systemic lupus erythematosus; T1DM: type 1 diabetes mellitus
