# Supplementary material for: Risks of autoimmune and inflammatory post-acute COVID-19 conditions: a network cohort study in six European countries, the USA and Korea
Source: BMJ Public Health. 2026 Jul 24;4(3):e001686. doi: 10.1136/bmjph-2024-001686 (PMC13404851; doi:10.1136/bmjph-2024-001686)
Supplement: online supplemental table 6 [file bmjph-4-3-s015.docx]

#### Supplementary Table 6. Input data for the meta-analyses of incidence rate ratios of reinfection versus previous COVID-19

| **Person-years exposed** | **# events exposed** | **IR exposed per**  **100’000 py** | **Person-years unexposed** | **# events unexposed** | **IR un-exposed per 100’000 py** | **database** | **Age group [years]** | **sex** | **outcome** |
| --- | --- | --- | --- | --- | --- | --- | --- | --- | --- |
| 1978.677618 | <5 | NA | 147246.8583 | 55 | 37.35224 | CPRD GOLD | 0 to 150 | Both | IBD |
| 852.881588 | <5 | NA | 7746.223135 | <5 | NA | IMASIS | 0 to 150 | Both | IBD |
| NA | NA | NA | 104516.011 | 147 | 140.6483 | P+ | 0 to 150 | Both | IBD |
| 6426.527036 | <5 | NA | 114135.9261 | 54 | 47.31201 | IPCI | 0 to 150 | Both | IBD |
| 707.9890486 | <5 | NA | 26838.89391 | 20 | 74.51872 | CORIVA | 0 to 150 | Both | IBD |
| 3250.852841 | <5 | NA | 475687.8029 | 265 | 55.70881 | CPRD Aurum | 0 to 150 | Both | IBD |
| 29526.94045 | <5 | NA | 371537.9658 | 36 | 9.689454 | NLHR@UIO | 0 to 150 | Both | IBD |
| NA | NA | NA | 147266.0616 | <5 | NA | CPRD GOLD | 0 to 150 | Both | Juvenile arthritis |
| NA | NA | NA | 104559.091 | 5 | 4.781985 | P+ | 0 to 150 | Both | Juvenile arthritis |
| 6427.5154 | <5 | NA | 114154.8501 | <5 | NA | IPCI | 0 to 150 | Both | Juvenile arthritis |
| NA | NA | NA | 26846.77892 | <5 | NA | CORIVA | 0 to 150 | Both | Juvenile arthritis |
| NA | NA | NA | 475774.2478 | 2 | NA | CPRD Aurum | 0 to 150 | Both | Juvenile arthritis |
| 29527.24435 | <5 | NA | 371544.7228 | 19 | 5.113785 | NLHR@UIO | 0 to 150 | Both | Juvenile arthritis |
| 1978.228611 | <5 | NA | 147238.0945 | 76 | 51.61708 | CPRD GOLD | 0 to 150 | Both | ME/CFS diagnosis |
| 854.0260096 | <5 | NA | 7746.212183 | 7 | 90.36675 | IMASIS | 0 to 150 | Both | ME/CFS diagnosis |
| 1337.360712 | 14 | 1046.838 | 104296.8214 | 803 | 769.918 | P+ | 0 to 150 | Both | ME/CFS diagnosis |
| 6426.171116 | <5 | NA | 114150.8172 | 13 | 11.38844 | IPCI | 0 to 150 | Both | ME/CFS diagnosis |
| NA | NA | NA | 3941.880903 | <5 | NA | CHUM | 0 to 150 | Both | ME/CFS diagnosis |
| 707.9890486 | <5 | NA | 26844.50103 | 12 | 44.70189 | CORIVA | 0 to 150 | Both | ME/CFS diagnosis |
| NA | NA | NA | 475721.6564 | 144 | 30.2698 | CPRD Aurum | 0 to 150 | Both | ME/CFS diagnosis |
| 29525.11978 | 8 | 27.09557 | 371518.2204 | 97 | 26.10908 | NLHR@UIO | 0 to 150 | Both | ME/CFS diagnosis |
| 1976.172485 | 23 | 1163.866 | 146934.59 | 1182 | 804.4396 | CPRD GOLD | 0 to 150 | Both | ME/CFS symptoms |
| 851.0362765 | 14 | 1645.053 | 7729.163587 | 94 | 1216.173 | IMASIS | 0 to 150 | Both | ME/CFS symptoms |
| 1326.436687 | 65 | 4900.347 | 102882.9569 | 5340 | 5190.364 | P+ | 0 to 150 | Both | ME/CFS symptoms |
| 6372.793977 | 176 | 2761.74 | 113154.5626 | 2933 | 2592.03 | IPCI | 0 to 150 | Both | ME/CFS symptoms |
| NA | NA | NA | 3941.371663 | <5 | NA | CHUM | 0 to 150 | Both | ME/CFS symptoms |
| 704.4161533 | 17 | 2413.346 | 26729.02122 | 353 | 1320.662 | CORIVA | 0 to 150 | Both | ME/CFS symptoms |
| 3243.348392 | 45 | 1387.455 | 473678.7598 | 6761 | 1427.339 | CPRD Aurum | 0 to 150 | Both | ME/CFS symptoms |
| 29384.97467 | 451 | 1534.798 | 368418.5927 | 8592 | 2332.13 | NLHR@UIO | 0 to 150 | Both | ME/CFS symptoms |
| NA | NA | NA | 104560.6051 | <5 | NA | P+ | 0 to 150 | Both | MIS |
| NA | NA | NA | 26846.71869 | <5 | NA | CORIVA | 0 to 150 | Both | MIS |
| NA | NA | NA | 475775.5729 | <5 | NA | CPRD Aurum | 0 to 150 | Both | MIS |
| 1978.710472 | <5 | NA | 147234.4093 | 112 | 76.06917 | CPRD GOLD | 0 to 150 | Both | POTS diagnosis |
| 853.4537988 | <5 | NA | 7743.101985 | 23 | 297.0386 | IMASIS | 0 to 150 | Both | POTS diagnosis |
| 1334.12731 | 35 | 2623.438 | 104124.3997 | 1406 | 1350.308 | P+ | 0 to 150 | Both | POTS diagnosis |
| 6426.223135 | <5 | NA | 114126.412 | 81 | 70.97393 | IPCI | 0 to 150 | Both | POTS diagnosis |
| NA | NA | NA | 3938.718686 | 8 | 203.1117 | CHUM | 0 to 150 | Both | POTS diagnosis |
| 707.1759069 | 5 | 707.0377 | 26816.32033 | 93 | 346.8037 | CORIVA | 0 to 150 | Both | POTS diagnosis |
| 3249.327858 | 7 | 215.4292 | 475549.6756 | 729 | 153.2963 | CPRD Aurum | 0 to 150 | Both | POTS diagnosis |
| 29524.97741 | 10 | 33.86963 | 371445.5469 | 307 | 82.65007 | NLHR@UIO | 0 to 150 | Both | POTS diagnosis |
| 1974.360027 | 25 | 1266.233 | 146832.3012 | 1552 | 1056.988 | CPRD GOLD | 0 to 150 | Both | POTS symptoms |
| 851.7207392 | 11 | 1291.503 | 7723.917864 | 95 | 1229.946 | IMASIS | 0 to 150 | Both | POTS symptoms |
| 1323.099247 | 79 | 5970.829 | 102554.0534 | 6309 | 6151.878 | P+ | 0 to 150 | Both | POTS symptoms |
| 6349.445585 | 233 | 3669.612 | 112692.6489 | 4309 | 3823.674 | IPCI | 0 to 150 | Both | POTS symptoms |
| NA | NA | NA | 3936.698152 | 13 | 330.226 | CHUM | 0 to 150 | Both | POTS symptoms |
| 703.2197125 | 23 | 3270.671 | 26683.98084 | 467 | 1750.114 | CORIVA | 0 to 150 | Both | POTS symptoms |
| 3237.869952 | 59 | 1822.186 | 473086.3847 | 8350 | 1765.005 | CPRD Aurum | 0 to 150 | Both | POTS symptoms |
| 29332.3258 | 616 | 2100.072 | 366850.9541 | 12680 | 3456.445 | NLHR@UIO | 0 to 150 | Both | POTS symptoms |
| NA | NA | NA | 147254.6092 | 40 | 27.16384 | CPRD GOLD | 0 to 150 | Both | RA |
| NA | NA | NA | 7745.149897 | 7 | 90.37914 | IMASIS | 0 to 150 | Both | RA |
| 1340.5859 | <5 | NA | 104504.8652 | 164 | 156.9305 | P+ | 0 to 150 | Both | RA |
| 6426.075291 | <5 | NA | 114130.6146 | 65 | 56.95229 | IPCI | 0 to 150 | Both | RA |
| 707.926078 | <5 | NA | 26838.1629 | 25 | 93.15094 | CORIVA | 0 to 150 | Both | RA |
| 3251.041752 | <5 | NA | 475721.024 | 170 | 35.73523 | CPRD Aurum | 0 to 150 | Both | RA |
| 29521.29774 | 20 | 67.7477 | 371390.2697 | 421 | 113.3578 | NLHR@UIO | 0 to 150 | Both | RA |
| NA | NA | NA | 147265.3032 | <5 | NA | CPRD GOLD | 0 to 150 | Both | SLE |
| NA | NA | NA | 7746.499658 | <5 | NA | IMASIS | 0 to 150 | Both | SLE |
| NA | NA | NA | 104551.4004 | 26 | 24.86815 | P+ | 0 to 150 | Both | SLE |
| NA | NA | NA | 3941.979466 | <5 | NA | CHUM | 0 to 150 | Both | SLE |
| 3250.896646 | <5 | NA | 475772.0219 | 12 | 2.522216 | CPRD Aurum | 0 to 150 | Both | SLE |
| NA | NA | NA | 371550.475 | <5 | NA | NLHR@UIO | 0 to 150 | Both | SLE |
| NA | NA | NA | 147259.4579 | 26 | 17.65591 | CPRD GOLD | 0 to 150 | Both | T1DM |
| NA | NA | NA | 7747.080082 | <5 | NA | IMASIS | 0 to 150 | Both | T1DM |
| 1340.260096 | <5 | NA | 104538.2122 | 70 | 66.96116 | P+ | 0 to 150 | Both | T1DM |
| 6427.263518 | <5 | NA | 114149.807 | 17 | 14.89271 | IPCI | 0 to 150 | Both | T1DM |
| NA | NA | NA | 3940.517454 | <5 | NA | CHUM | 0 to 150 | Both | T1DM |
| 707.5427789 | <5 | NA | 26843.9206 | 8 | 29.80191 | CORIVA | 0 to 150 | Both | T1DM |
| NA | NA | NA | 475743.9973 | 98 | 20.59931 | CPRD Aurum | 0 to 150 | Both | T1DM |
| 29526.00411 | <5 | NA | 371491.1759 | 158 | 42.53129 | NLHR@UIO | 0 to 150 | Both | T1DM |

IBD: inflammatory bowel disease; ME/CFS: myalgic encephalomyelitis / chronic fatigues syndrome; POTS: postural orthostatic tachycardia syndrome; RA: rheumatoid arthritis; SLE: systemic lupus erythematosus; T1DM: type 1 diabetes mellitus
